# Paxbp1 is indispensable for the survival of CD4 and CD8 double-positive thymocytes

**DOI:** 10.3389/fimmu.2023.1183367

**Published:** 2023-06-19

**Authors:** Wenting Li, Yang Yang, Shenglin Liu, Dongsheng Zhang, Xuanyao Ren, Mindan Tang, Wei Zhang, Xiaofan Chen, Cong Huang, Bo Yu

**Affiliations:** ^1^ Department of Dermatology, Peking University Shenzhen Hospital, Shenzhen, Guangdong, China; ^2^ Shenzhen Key Laboratory for Translational Medicine of Dermatology, Shenzhen Peking University - The Hong Kong University of Science and Technology Medical Center, Shenzhen, Guangdong, China; ^3^ Shenzhen Key Laboratory for Translational Medicine of Dermatology, Biomedical Research Institute, Shenzhen Peking University - The Hong Kong University of Science and Technology Medical Center, Shenzhen, Guangdong, China; ^4^ Key Laboratory of Research and Utilization of Ethnomedicinal Plant Resources of Hunan Province, College of Biological and Food Engineering, Huaihua University, Huaihua, Hunan, China; ^5^ Shenzhen Bay Laboratory, Shenzhen, Guangdong, China

**Keywords:** Paxbp1, apoptosis, double-positive (DP) thymocytes, development, thymus

## Abstract

The lifespan of double-positive (DP) thymocytes is critical for intrathymic development and shaping the peripheral T cell repertoire. However, the molecular mechanisms that control DP thymocyte survival remain poorly understood. Paxbp1 is a conserved nuclear protein that has been reported to play important roles in cell growth and development. Its high expression in T cells suggests a possible role in T cell development. Here, we observed that deletion of Paxbp1 resulted in thymic atrophy in mice lacking Paxbp1 in the early stages of T cell development. Conditional loss of Paxbp1 resulted in fewer CD4^+^CD8^+^ DP T cells, CD4 and CD8 single positive (SP) T cells in the thymus, and fewer T cells in the periphery. Meanwhile, Paxbp1 deficiency had limited effects on the CD4^-^CD8^-^ double negative (DN) or immature single-positive (ISP) cell populations. Instead, we observed a significant increase in the susceptibility of Paxbp1-deficient DP thymocytes to apoptosis. Consistent with this, RNA-Seq analysis revealed a significant enrichment of the apoptotic pathway within differentially expressed genes in Paxbp1-deficient DP cells compared to control DP cells. Together, our results suggest a new function for Paxbp1, which is an important mediator of DP thymocyte survival and critical for proper thymic development.

## 1 Introduction

T cell development is critical for T cell-mediated cellular immunity (1). T cell development proceeds through a finely tuned cellular program. First, bone marrow (BM)-derived lymphoid progenitor cells enter the thymus to initiate differentiation into mature T cells (2). In the thymus, CD4^-^CD8^-^ double negative (DN) thymocytes undergo sequential DN1-4 stages, entailing pre-TCR complex formation and several cycles of proliferation (3). Then, DN4-stage cells become immature single-positive (ISP) cells, followed by up-regulation of CD4 expression and then differentiation into CD4^+^CD8^+^ double-positive (DP) cells (4). Only a small percentage of DP cells survive through the positive and negative selection processes to become mature CD4^+^ or CD8^+^ SP cells (5). Then they move into peripheral lymphoid organs, where they play crucial roles in adaptive immunity (6).

The lifespan of DP thymocytes shapes the peripheral T cell repertoire, which is essential for mounting immune responses against foreign antigens (7). Given the importance of DP thymocyte survival, a thorough understanding of the mechanisms whereby DP thymocytes evade apoptosis is necessary. Previous evidence showed that several factors, such as RORγt (Retinoid-related orphan nuclear factor γt) (8, 9), TCF-1 (T cell factor-1) (10, 11), c-Myb (myeloblastosis oncogene) (12), and HEB (13, 14) influence thymocyte survival *via* a Bcl-xL-dependent pathway. However, the precise molecular mechanisms that control DP thymocyte survival remain poorly understood.

Paxbp1 is a widely-expressed nuclear protein that can be found in a variety of tissue types and cells, including muscles, immune cells, and neurons (15). A growing body of work suggests an important role for Paxbp1 in controlling the growth and development of multiple cells and tissues (16). In mice, Paxbp1 gene deletion causes embryonic mortality and various developmental defects (16). In addition, Paxbp1 was identified as one of the most significantly dysregulated genes in Ts1Cje (a mouse model for Down syndrome) postnatal brain development (17). Clinically, a potentially harmful variation in the Paxbp1 gene was linked to hypotonia and developmental delay (18). It was recently reported that Paxbp1 is closely associated with COVID-19 disease severity, demonstrating its possible role in immune modulation (19). Thus, Paxbp1 is instrumental in multiple developmental processes and disorders. However, the exact physiological functions of Paxbp1 remain to be established.

Given the high levels of Paxbp1 expression in T cells and its crucial role in development, we hypothesized that Paxbp1 may also influence the thymocyte development. Here, we found that Lck-cre-mediated T cell-specific loss of Paxbp1 (abbreviated as Paxbp1 cKO) resulted in thymic atrophy and significant reductions in the numbers of DP cells, CD4^+^ SP, and CD8^+^ SP cells in the thymus, and fewer T cells in the periphery. The DN and ISP cell populations appeared normal in the Paxbp1 cKO mice, whereas the DP thymocytes of Paxbp1 cKO mice were much more susceptible to apoptosis than those in the control mice. Further RNA-seq analysis showed a significant enrichment of the apoptosis pathway within differentially-expressed genes between Paxbp1 cKO and control mice. Taken together, our results offer an important missing piece to the puzzle of Paxbp1 function in the regulatory mechanism of intrathymic development: the presence of Paxbp1 in the thymus is critical for maintaining T cell survival.

## 2 Materials and methods

### 2.1 Mice

The Paxbp1^fl/fl^ mice on the C57BL/6 background were generously provided by Professor Wu from Hong Kong University of Science and Technology (16). The B6.Cg-Tg (Lck-cre) 548Jxm/J Lck-cre mice were obtained from the Jackson Laboratory. A conditional deletion of Paxbp1 in T cells was generated by cross-breeding Paxbp1^fl/fl^ mice with Lck-cre mice. Mice used in this study were 4–7 weeks of age and mixed sexes. Littermate Paxbp1^fl/fl^ mice were used as controls in all experiments. Animals were maintained under specific pathogen-free conditions. All the animal experiments were approved by the Committee for the Ethics of Animal Experiments, Shenzhen Peking University‐The Hong Kong University of Science and Technology Medical Center (SPHMC). Mice were genotyped using the following primers: Paxbp1 Floxed allele was detected by PCR using the Flox forward primer: 5’-GGGTACTTTATATGAGTGAGAGGC-3’ and reverse primer: 5’-AGGTAAATGTCCAGTGCCTG -3’. The Lck-cre transgene was detected by PCR using the Lck-cre forward primer: 5’-TGTGAACTTGGTGCTTGAGG-3’ and reverse primer: 5’-CAGGTTCTTGCGAACCTCAT-3’.

### 2.2 H&E staining and immunohistochemistry staining

Mouse thymus and spleen were isolated and formalin-fixed, followed by embedding in paraffin. Paraffin sections were used for routine H&E staining. CD4 (Abcam, ab183685) levels were detected using immunohistochemistry as described previously (20).

### 2.3 Flow cytometry staining and cell sorting

Single-cell suspensions were prepared from the thymus and spleens of Paxbp1 cKO and control mice. For cell surface marker staining, 2 million cells were incubated with the fluorescent antibody mixture at 4°C for 30 min, washed with PBS, and then analyzed by FACS. For intracellular staining, cells were stained with appropriate surface markers, then fixed and stained with antibodies according to the manufacturer’s protocol for the Cyto-Fast™ Fix/Perm Buffer Set (BioLegend, 428703). Zombie Violet™ Fixable Viability Kit (Biolegend, 423113) or 7-AAD (BD, 420404) was used to mark dead cells. The Backman Coulter CytoFLEX S (Backman) was used for the flow cytometric analysis. For cell sorting, thymocytes were isolated and stained with surface markers as indicated above. Then labeled cells were resuspended in FACS buffer (1% FBS + 2 mM EDTA, 25 mM HEPES in PBS). DN, ISP, DP, and SP thymocytes were sorted on an FACSAria III (BD Biosciences).

The following antibodies were used: anti-CD4 (RM-5), anti-CD8 (53–6.7), anti-TCRβ (H57–597), anti-CD44 (IM7), anti-CD25 (PC61), anti-TCRγ/δ (GL3), anti-CD5 (53–7.3), anti-CD69 (H1.2F3), and anti-CD19 (6D5), all of which were purchased from BioLegend. Anti-CD62L (MEL-14) was purchased from Invitrogen.

### 2.4 *In vitro* assay of ISP development

ISP thymocytes were isolated from Paxbp1 cKO mice and control mice by FACS. Equal numbers of ISP cells were incubated for 16 hours in an ISP culture medium (RPMI 1640 medium supplemented with 10% FBS, 1% penicillin and streptomycin, 1× MEM non-essential amino acids, 1× sodium pyruvate, and 55 nM 2-mercaptoethanol). After that, the cells were harvested and analyzed for CD4 and CD8 surface expression.

### 2.5 TUNEL

Thymuses from control and Paxbp1 cKO mice were fixed in 4% paraformaldehyde. The paraffin-embedded sections (3 μm) were deparaffinized and dehydrated. The TUNEL assay was performed using a Colorimetric TUNEL Apoptosis Assay kit (Beyotime, C1098) according to the manufacturer’s protocol.

### 2.6 Apoptosis *in vivo*

Thymocytes were freshly isolated from control and Paxbp1 cKO mice. Cells were stained with surface markers (CD4, CD8, and TCRβ) at room temperature for 20 min, then washed with PBS. Annexin V (BD, 559763) staining and caspase-3 staining (Beyotime, C1168M) were carried out according to the manufacturers’ instructions, respectively. Then, cell apoptosis was analyzed by flow cytometry.

For anti-CD3 antibody stimulation, mice were intraperitoneally (i.p.) injected with anti-CD3 antibody (eBioscience, 100340, clone: 145-2C11) (10 μL per mouse). 24 hours later, mice were euthanized, and the thymuses were freshly isolated from control and Paxbp1 cKO mice. Annexin V and caspase-3 staining were then performed according to the manufacturers’ instructions.

### 2.7 Apoptosis *in vitro*

Thymocytes (2×10^6^) were cultured overnight in 96-well plates. Then cells were stained with surface markers (CD4 and CD8) at 4 °C for 30 min, washed with cold PBS, then stained with annexin V and caspase-3 as described above.

### 2.8 BrdU assay

Paxbp1 cKO mice and control mice were injected with 1 mg of BrdU i.p. Thymus cells were harvested an hour later. Thymocytes (2×10^6^) were labeled with surface markers. Subsequently, BrdU incorporation was detected with the FITC BrdU Flow Kit (BD Biosciences) according to the manufacturer’s protocol.

### 2.9 RNA sequencing and data analysis

Total RNA was extracted from DP thymocytes using the RNeasy Kit (Qiagen, 74004) according to the manufacturer’s protocol. Purified RNA was quantified using a NanoDrop 2000 (Thermo Scientific) and the RNA Nano 6000 Assay Kit of the Bioanalyzer 2100 system (Agilent Technologies, CA, USA). All the RNA samples had an RNA Quality Index ≥8. Next, cDNA synthesis and pre-amplification of cDNAs were performed using the Discover-sc WTA Kit V2 (Vazyme) according to the manufacturer’s protocol. And then the cDNAs were purified with VAHTS DNA Clean Beads. Sequencing libraries were constructed as described in the protocol of TruePrepTM DNA Library Prep Kit V2 (Vazyme #TD503). Lastly, library quality was assessed on an Agilent 2100 Bioanalyzer (Agilent Technologies, CA, USA) and sequenced using Illumina HiSeq2500 by Gene Denovo Biotechnology Co. (Guangzhou, China).

The DEseq2 software was used to detect differentially expressed genes (DEGs) between different groups. Differentially expressed genes with an absolute log2 fold change > 1 and FDR < 0.05 were considered significant. Kyoto Encyclopedia of Genes and Genomes (KEGG) pathway enrichment analysis was implemented using the cluster Profiler R package. We performed gene set enrichment analysis using the software GSEA and MSigDB to identify whether a set of genes in specific KEGG pathways shows significant differences between two groups.

All sequencing data have been submitted to the NCBI Gene Expression Omnibus (GEO; http://www.ncbi.nlm.nih.gov/geo/) under accession number GSE233211.

### 2.10 Real-time quantitative PCR

Thymocytes were sorted as described above, and total RNA was extracted using the RNeasy Kit (Qiagen). Reverse transcription was performed using HiScript III RT SuperMix for quantitative PCR from Vazyme (R323-01). Real-time PCR was performed on the CFX96 Touch Real-Time PCR Detection System (Bio-Rad) using SYBR Green Supermix (Bio-Rad). The results were quantified using 2^-△△Ct^ method. Results were normalized to Gapdh expression.

### 2.11 Western blot analysis

The separated T cells were washed with PBS before RIPA lysis buffer was added. Then equal amounts of protein were electrophoretically separated on 10% gels and transferred onto PVDF membranes. The membranes were blocked with TBST containing 5% BSA. Western blot analysis was carried out using Paxbp1 (Proteintech, 21357-1-AP). According to the primary antibodies, the membranes were then incubated with appropriate horseradish peroxidase (HRP)-conjugated secondary antibodies. The immunosignal images were captured using the Bio-Rad imaging system and analyzed using the Image lab software from Bio-Rad.

### 2.12 Statistical analysis

Statistical analysis was performed using GraphPad Prism Software 8 (GraphPad). The data were analyzed by the student t test and shown as mean ± S.D. The p value was used to determine whether an effect was significant (* p < 0.05, ** p < 0.01; *** p < 0.001; **** p < 0.0001).

## 3 Results

### 3.1 Stage-specific deletion of Paxbp1 in thymocytes

**Figure 1:**
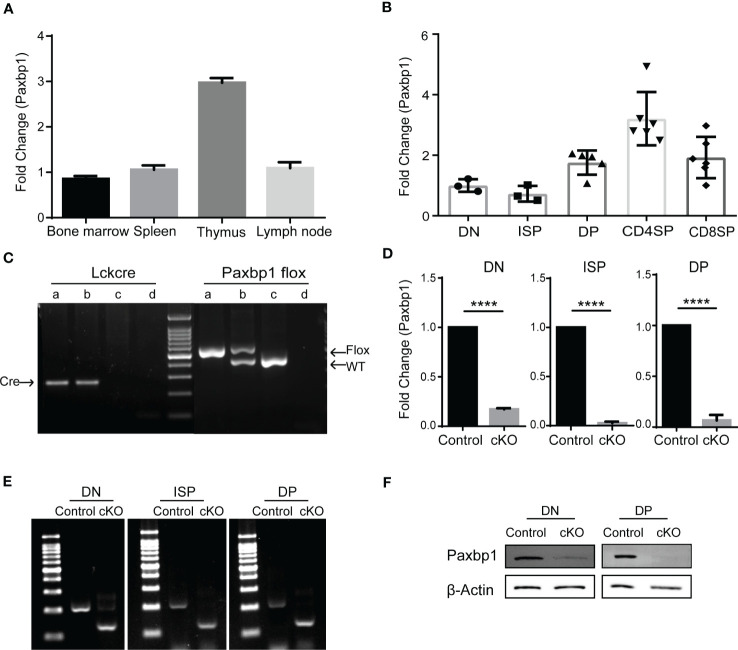
Conditional deletion of Paxbp1 in T cells. **(A)** *Paxbp1* mRNA levels in different organs. The mRNA levels of *Paxbp1* in C57BL/6 organs were evaluated by qPCR analysis (n = 3). **(B)** qPCR detection of the *Paxbp1* mRNA levels in different T cell subsets, including DN (CD4^-^CD8^-^), ISP (CD4^-^CD8^+^TCRβ^-^), DP (CD4^+^CD8^+^), CD4 SP (CD4^+^CD8^-^TCRβ^+^), and CD8 SP (CD4^-^CD8^+^TCRβ^+^) (n > 3). **(C)** Genotyping of Paxbp1 conditional knockout mice. Sample a represents the Paxbp1^fl/fl^ Lck-cre^+^ cKO mouse; sample b represents the Paxbp1^fl/-^ Lck-cre^+^ mouse; sample c the represents WT mouse; sample d is the negative control (H_2_O). **(D)** The knockout efficiency of Paxbp1^fl/fl^ Lck-cre mice. RNA isolated from DN, ISP, and DP T cell subsets was assessed for *Paxbp1* expression by qPCR (n > 3). **(E)** Agarose gel image of PCR products in DN, ISP, and DP cells from control and cKO mice. Total RNA extracted from sorted DN, ISP, and DP cells was subjected to reverse transcription PCR, followed by the PCR amplification to detect the deleted allelic sequences. **(F)** Western blot analysis for detection of Paxbp1 levels in sorted DN and DP thymocytes. The p value was used to determine whether an effect was significant (**** p < 0.0001).

Paxbp1 is broadly expressed in a variety of tissues and cells (15). In immune tissues, we detected a high mRNA level of *Paxbp1* in the thymus, where T cells are abundant (**Figure 1A**). We also measured *Paxbp1* expression in different T-cell subsets. As shown in **Figure 1B**, *Paxbp1* was highly expressed in DP thymocytes, especially in CD4 SP thymocytes. Consistent with these results, the mouse and human expression databases further confirmed that the expression pattern of Paxbp1 is conserved across species (**Supplementary Figure 1**). Collectively, these findings suggest a requirement for Paxbp1 in T-cell development.

To investigate the physiological role of Paxbp1 in T cells, we crossed mice carrying a conditional Paxbp1 allele (Paxbp1^fl/fl^) in exons 8 flanked by loxp sites with Lck-cre transgenic mice to specifically delete Paxbp1 in T cells during mouse development. Mice were genotyped to confirm conditional knockout and to identify littermate Paxbp1^fl/fl^ mice, which were used as controls (**Figure 1C**). As shown in **Figures 1D, E**, *Paxbp1* levels were markedly reduced in DN thymocytes in the Paxbp1 cKO mice relative to the control mice; deletion was complete by the ISP stage. Meanwhile, western blot analysis of the sorted DN and DP thymocytes from Paxbp1 cKO mice showed efficient deletion of the Paxbp1 protein compared with the control mice (**Figure 1F**). Therefore, we expected that the Paxbp1 cKO mice would allow us to investigate the function of Paxbp1 in early T cell development.

### 3.2 The ablation of Paxbp1 at the early development stage results in aberrant thymocyte development

**Figure 2:**
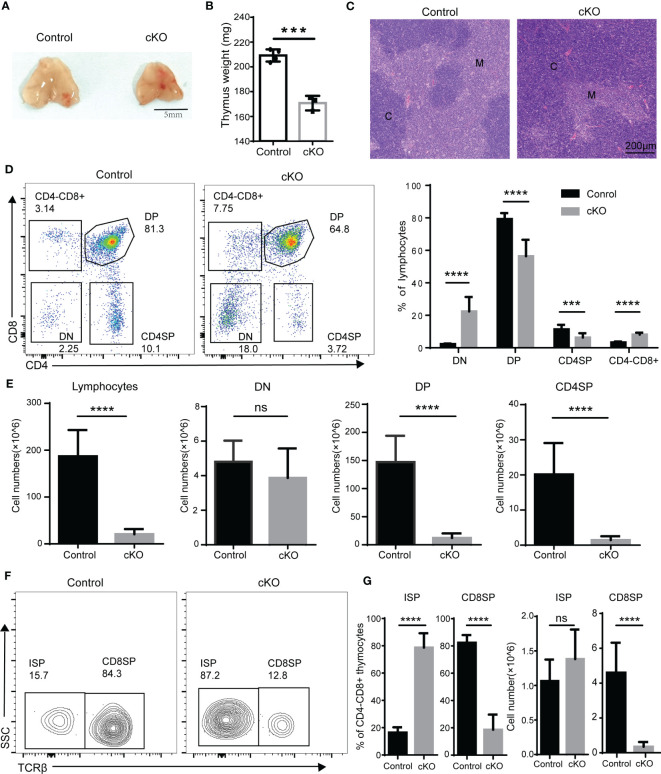
Defective T cell development in Paxbp1 cKO mice. **(A)** Representative pictures showing the thymus from control and Paxbp1 cKO mice (Scale bar = 5 mm). **(B)** The weights of the thymus in the control and Paxbp1 cKO mice (n = 3). **(C)** Hematoxylin- and eosin-stained sections of the control and Paxbp1 cKO thymus (C: cortex; M: medulla; scale bar = 200 μm). **(D)** Cell surface staining of CD4 and CD8 on the control and Paxbp1 cKO thymus (number in quadrants indicates percentage of cells in each throughout). Bar charts indicate the frequency of the different stages of thymocyte development in control and Paxbp1 cKO mice (n = 18 for control mice, n = 13 for Paxbp1 cKO mice). **(E)** Bar charts indicate the cell numbers of the different stages of thymocyte development in control and Paxbp1 cKO mice (n = 18 for control mice, n = 13 for Paxbp1 cKO mice). **(F)** TCRβ staining is shown for pre-gated CD4^+^CD8^−^ thymocytes in the control and Paxbp1 cKO mice. **(G)** Bar charts show the frequency and cell numbers of ISP cells and CD8 cells. (n > 10 for each genotype). The p value was used to determine whether an effect was significant (*** p < 0.001; **** p < 0.0001, ns means no significant).

We next analyzed thymocyte development in Paxbp1 cKO mice. Significant reductions in both thymus size (**Figure 2A**) and thymus weight (**Figure 2B**) were observed in Paxbp1 cKO mice compared with the control mice. Histological analysis revealed a pronounced cortical expansion and a rudimentary medulla in the Paxbp1 cKO thymus (**Figure 2C**), suggesting that Paxbp1 deletion leads to thymic atrophy in mice. Additionally, the early loss of Paxbp1 at the DN stage was accompanied by an aberrant distribution of thymocytes among the four subpopulations: the percentage of DP and CD4SP was decreased, whereas the percentages of DN and CD4^-^CD8^+^ thymocytes were markedly increased (**Figure 2D**). In terms of absolute cell numbers, a similar number of DN thymocytes and substantially fewer DP and CD4SP thymocytes were found in Paxbp1 cKO mice (**Figure 2E**).

Within the CD4^-^CD8^+^ population, a considerable subset of Paxbp1-depleted cells did not express surface TCR compared with control cells, identifying them as ISP thymocytes (**Figure 2F**). Despite a notable increase in the proportion of ISP cells detected in Paxbp1 cKO mice, the number of ISPs was comparable to that of the control mice (**Figure 2G**). However, both the percentage and the absolute cell number of CD8 SP cells decreased (**Figure 2G**). Thus, Paxbp1 deficiency led to the loss of DP and SP thymocytes but not DN and ISP thymocytes.

**Figure 3:**
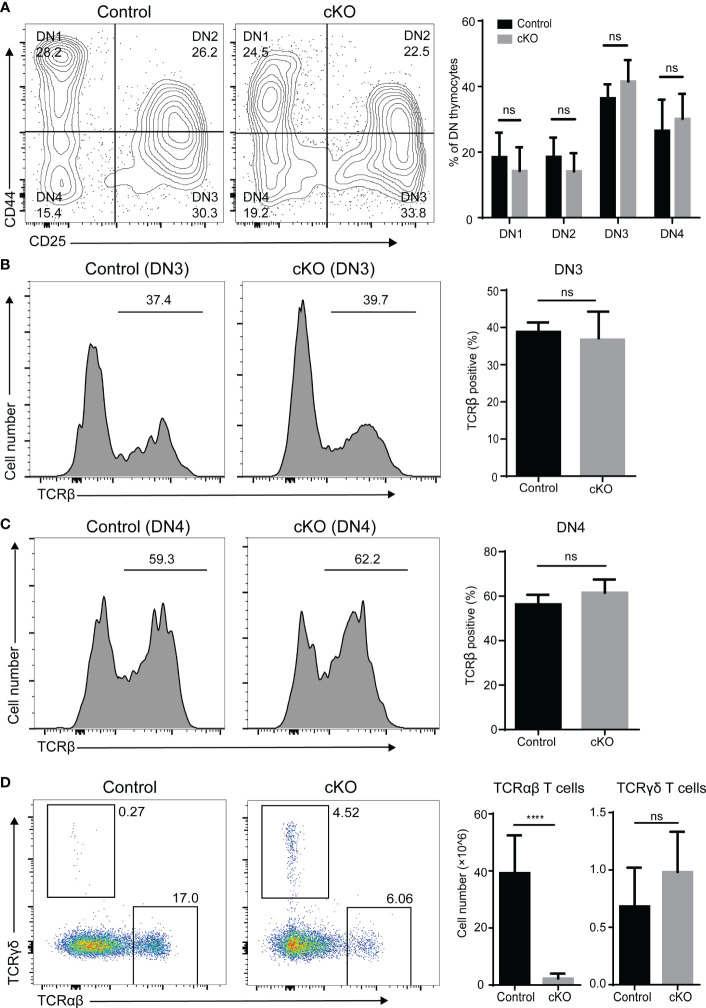
Phenotype of DN thymocytes in Paxbp1 cKO mice. **(A)** CD44 and CD25 staining is shown for pre-gated CD4^−^CD8^−^ thymocytes; the bar chart shows the quantification results (n = 8 for control mice, n = 6 for Paxbp1 cKO mice). **(B)** Intracellular TCRβ expression in DN3 thymocytes from the control and Paxbp1 cKO mice; the bar chart shows the quantification results (n = 7 for each genotype). **(C)** Intracellular TCRβ expression in DN4 thymocytes from the control and Paxbp1 cKO mice; the bar chart shows the quantification results (n = 7 for each genotype). **(D)** TCRβ and TCRδ staining is shown for total thymocytes in the control and Paxbp1 cKO mice. Bar charts indicate the frequency and cell numbers of αβ T and γδ T thymocytes in the control and Paxbp1 cKO mice (n = 11 for control mice, n = 6 for Paxbp1 cKO mice). The p value was used to determine whether an effect was significant (**** p < 0.0001; ns means no significant).

We further evaluated thymocyte differentiation in the DN compartment based on CD44 and CD25 expression. As expected, the frequencies of the DN1 to DN4 subpopulations showed little variation (**Figure 3A**). It is widely accepted that TCRβ gene rearrangement is necessary for DN cells to mature into ISP cells (21). We therefore examined intracellular TCR expression of DN3 and DN4 cells and did not find significant differences between Paxbp1 cKO mice and control mice (**Figures 3B, C**). Thus, deletion of Paxbp1 has little effect on the development of DN thymocytes.

Further analysis showed no difference in the cell number of γδ thymocytes between the Paxbp1 cKO mice and the control mice (**Figure 3D**). However, the cell number of αβ thymocytes was significantly decreased in Paxbp1 cKO mice (**Figure 3D**), suggesting a severe αβ-lineage specific developmental defect in Paxbp1 cKO thymocytes.

### 3.3 Paxbp1-deficient DP thymocytes are highly sensitive to apoptosis

**Figure 4:**
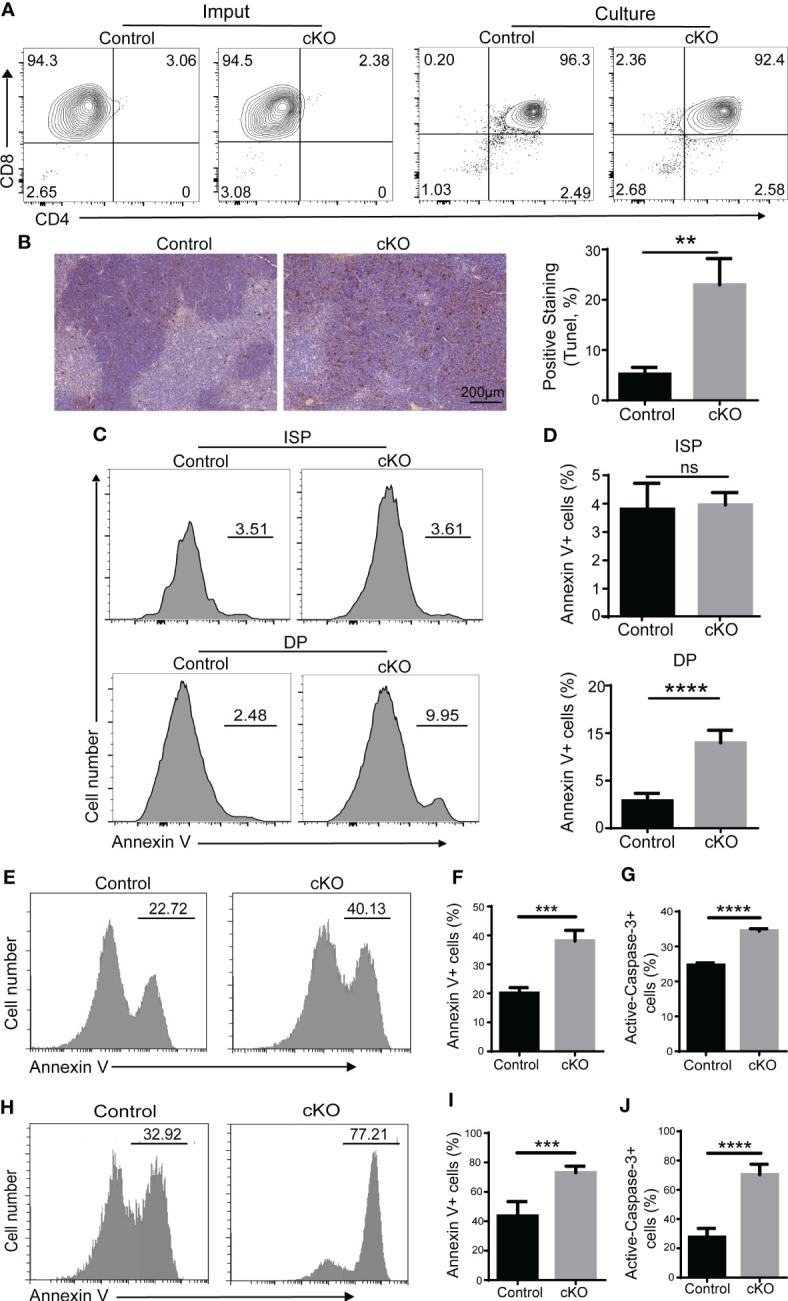
Impaired cell survival of Paxbp1 cKO thymocytes. **(A)** Maturation of ISP into DP (culture panels), relative to the imput (imput panels) of ISPs from the control and Paxbp1 cKO, was assessed by flow-cytometric measurement of CD4 and CD8 surface marker expression. **(B)** Staining of the total thymus with TUNEL from control and Paxbp1 cKO mice (Scale bar = 200 µm) and quantification of TUNEL positive thymocytes. **(C)** Annexin V staining of ISP and DP cells from control and Paxbp1 cKO mice. **(D)** The bar charts show the percentage of Annexin V-positive cells in ISP and DP thymocytes from control and Paxbp1 cKO mice (n > 4). **(E)** Annexin V staining of DP cells cultured overnight *in vitro*. **(F)** Percentages and quantification of annexin V-positive DP cells (n = 3). **(G)** The bar chart shows the quantification results of active caspase-3 staining of DP cells cultured overnight *in vitro* (n = 3). **(H)** Annexin V staining of DP cells from control and cKO mice *i.p.* injected with anti-CD3 antibody for 24 h. **(I)** Percentages and quantification of annexin V^+^ DP cells from control and cKO mice *i.p.* injected with anti-CD3 antibody for 24 h (n = 3). **(J)** The bar chart shows the quantification results of active caspase-3 staining in DP cells from control and cKO mice *i.p.* injected with anti-CD3 antibody for 24 h (n = 3). The p value was used to determine whether an effect was significant (** p < 0.01; *** p <0.001; **** p < 0.0001; ns means no significant).

Next, we focused on the function of Paxbp1 during the transition from the ISP to the DP period. ISP cells were sorted and cultured *in vitro*. As shown in **Figure 4A**, almost all of the ISP cells from Paxbp1 cKO mice matured into DP cells after an overnight culture, similar to those of the control mice. However, a higher proportion of apoptotic cells was detected in Paxbp1 cKO mice (data not shown), suggesting that Paxbp1 may affect the viability of the cells. Then we assessed the survival of Paxbp1-cKO and control thymocytes using the *in situ* TUNEL assay. As shown in **Figure 4B**, we detected an increase in TUNEL-positive cells in the Paxbp1 cKO thymus. Additionally, increased apoptosis of freshly separated DP thymocytes but not ISP thymocytes was observed in Paxbp1 cKO mice using Annexin V staining (**Figures 4C, D**).

It is widely accepted that the apoptosis detected *in vivo* or on freshly isolated thymocytes is usually very low because of the continuous clearance of apoptotic cells by macrophages (22). Therefore, thymocytes isolated from control and Paxbp1 cKO mice were cultured *in vitro* overnight before annexin V staining. We found that Paxbp1 deficiency led to a marked increase in apoptosis in DP thymocytes (**Figures 4E, F**), along with enhanced caspase-3 activity (**Figure 4G**).

We also analyzed the susceptibility of thymocytes from Paxbp1 cKO and control mice to cell death by *in vivo* i.p. administration of anti-CD3 antibody, which can induce rapid depletion of DP thymocytes (22). Increased annexin V labeling (**Figures 4H, I**) and enhanced caspase-3 activity (**Figure 4J**) in the DP thymocytes of Paxbp1 cKO mice were detected at 24 h after anti-CD3 injection. Thus, Paxbp1 deletion renders DP thymocytes highly susceptible to apoptosis.

### 3.4 Paxbp1 deficiency activates apoptosis-related pathways

**Figure 5:**
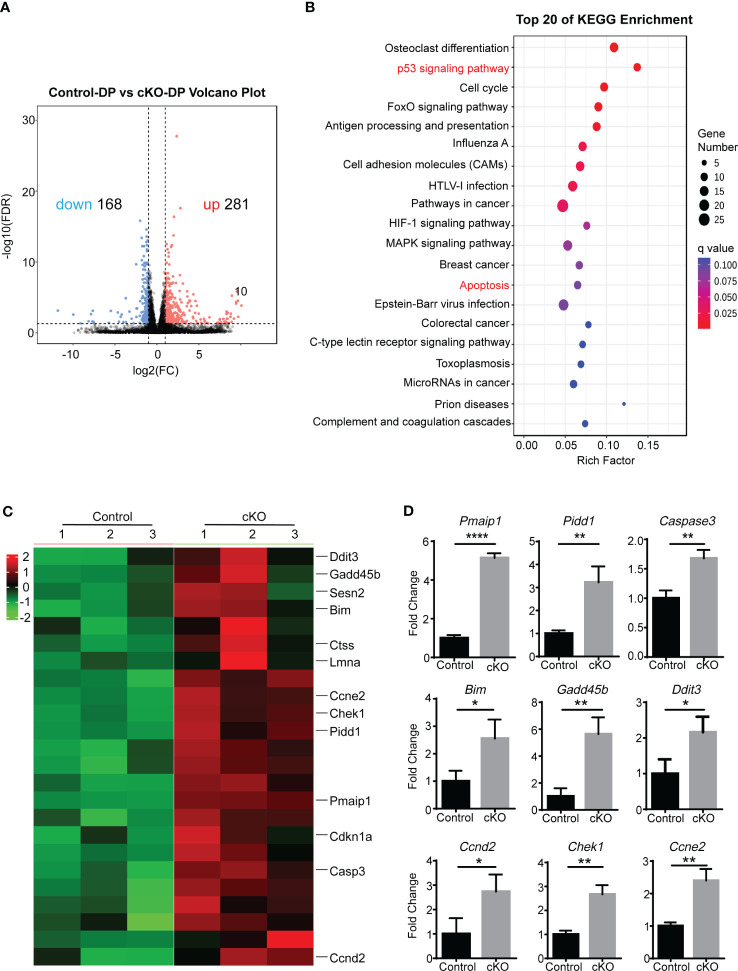
The RNA-Seq analysis of Paxbp1-deficient and control DP thymocytes. **(A)** Volcano plot of expression of all genes in Paxbp1-dificient and control DP thymocytes. Red, green, and black points represent genes that were upregulated, downregulated, and not significantly different in the mouse thymus respectively. X-axis: log2 ratio of gene expression levels, Y-axis: false-discovery rate values (-log10 transformed) of genes. **(B)** Kyoto Encyclopedia of Genes and Genomes (KEGG) pathway enrichment analysis for genes (change in expression of over 2-fold). The Y-axis label represents the pathway and the X-axis label represents the rich factor. The color and size of the bubble represent enrichment significance and the amount of differentially expressed genes enriched in the pathway, respectively. **(C)** Heatmap of representative genes related to p53 signaling pathway and apoptosis pathway. The scale ranges from minimum (green boxes) to medium (black boxes) to maximum (red boxes) relative expression. **(D)** qPCR analysis of some genes depicted in Heatmap (n=3). The p value was used to determine whether an effect was significant (* p < 0.05, ** p < 0.01; **** p < 0.0001).

In order to determine whether disregulation of a genetic pathway could explain the increased apoptosis of Paxbp1 cKO DP thymocytes, we performed high-throughput RNA sequencing (RNA-Seq) to compare gene transcription between control and Paxbp1-deficient DP cells. The analysis revealed 281 upregulated genes and 168 downregulated genes in Paxbp1-deficient DP thymocytes compared with control DP cells (**Figure 5A**).

Kyoto Encyclopaedia of Genes and Genomes (KEGG) pathways enriched two cell death-related pathways among the top 20 list: the p53 signaling pathway and the apoptosis pathway (**Figure 5B**). The differential expression of genes belonging to the two pathways was visualized in heatmap analysis (**Figure 5C**). Consistent with the RNA-seq data, qPCR results confirmed that the Paxbp1-deficient DP population displayed increased expression of *Casp3*, *Bim*, *Pmaip1*, *Pidd1*, etc. compared with that of the control group (**Figure 5D**). Taken together, the RNA-Seq and qPCR analyses demonstrated that Paxbp1 deficiency activated apoptosis-related pathways in DP thymocytes.

### 3.5 Increased cell cycle activity in Paxbp1-deficient DP thymocytes

**Figure 6:**
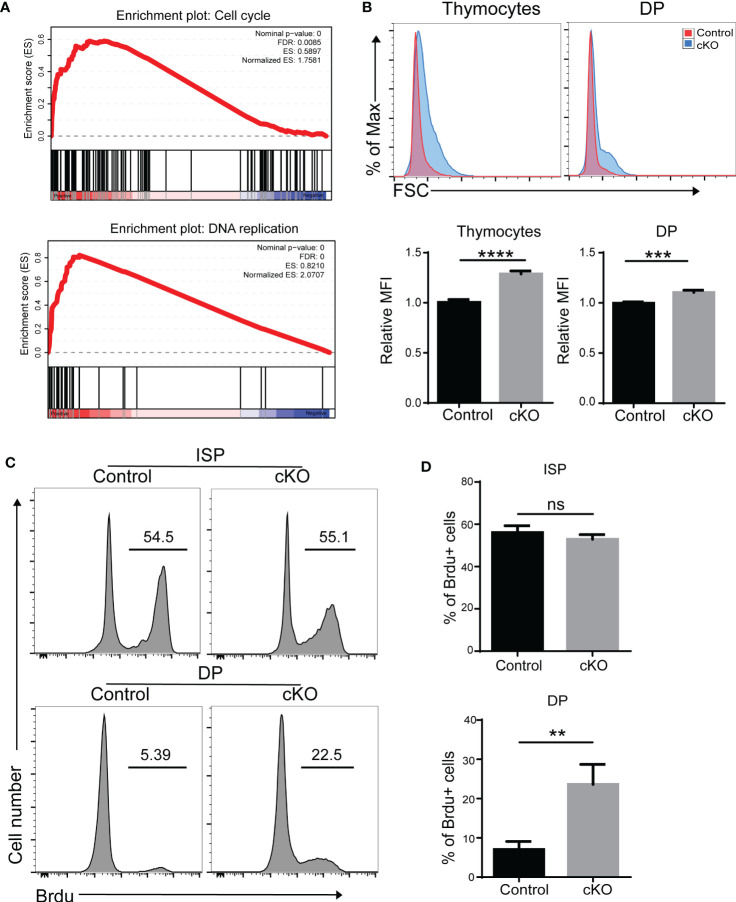
Increased cell cycle activity in Paxbp1-deficient DP thymocytes. **(A)** Gene set enrichment analysis (GSEA) reveals the up-representation of signature genes in the cell cycle pathway and DNA replication pathway in Paxbp1-deficient DP cells. **(B)** Cell size of total thymocytes and DP cells from control and Paxbp1 cKO mice. FSC, forward scattering. The bar charts show the quantification of the results. **(C)** BrdU incorporation in ISP, DP thymocytes of control and cKO mice. **(D)** Percentage of BrdU positive cells in ISP, DP thymocytes. The bar charts show the quantification of the results (n = 3). The p value was used to determine whether an effect was significant (** p < 0.01; *** p <0.001; **** p < 0.0001; ns means no significant).

Interestingly, we also found that cell cycle and DNA replication pathways were enriched in Paxbp1-deficient DP thymocytes (**Figure 6A**). We therefore measured the cell proliferation capacity of thymocytes. As shown in **Figure 6B**, thymocytes with higher forward scatter were seen in Paxbp1 cKO mice compared to the control mice. Moreover, the BrdU incorporation assay showed that DP cells, but not ISP cells, in Paxbp1 cKO mice exhibited increased cell proliferation compared with those in control mice (**Figures 6C, D**). Thus, reduced DP thymocyte cellularity in Paxbp1 cKO mice is not caused by a lower cell proliferation rate.

### 3.6 T-cell maturation appears normal in Paxbp1 cKO mice

**Figure 7:**
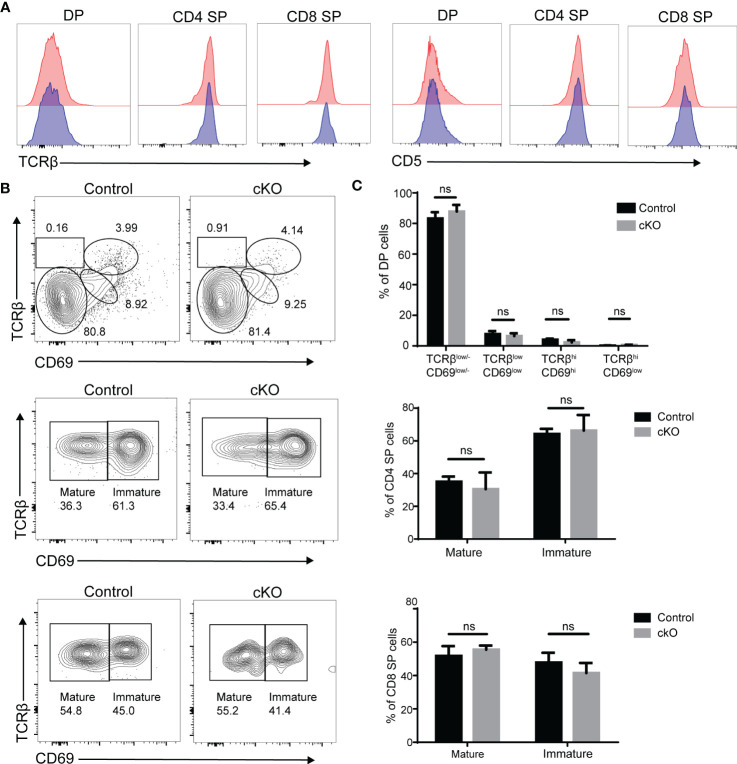
Unaltered expression of TCRβ, CD5 and CD69 in Paxbp1-deficient thymocytes. **(A)** Flow cytometry analysis of TCRβ and CD5 expression in DP, CD4 SP, and CD8 SP thymocytes from Paxbp1 cKO and control mice. Data are presented as representative half-overlay histograms of Paxbp1 cKO mice (blue) and control mice (red). **(B)** Gating on the DP population, DP development can be divided into four stages by TCRβ and CD69: Stage 1, TCRβ^low/-^CD69^low/-^; Stage 2, TCRβ^low^CD69^low^; Stage 3, TCRβ^hi^CD69^hi^; Stage 4, TCRβ^hi^CD69^low^ (up). Gating on the CD4 SP population: imature CD4 SP cells are TCRβ^hi^CD69^hi^, while mature CD4 SP cells are TCRβ^hi^CD69^low/-^ (medium). Gating on the CD8 SP population: imature CD8 SP cells are TCRβ^hi^CD69^hi^, while mature CD8 SP cells are TCRβ^hi^CD69^low/-^ (down). **(C)** Percentage and quantification of the results in **(B)** (n≥4). ns means no significant.

According to our results, DP thymocytes in Paxbp1 cKO mice were able to produce CD4 SP and CD8 SP cell populations, although the number of SP cells was substantially reduced. Signaling through the TCR complex on DP thymocytes regulates selection and results in upregulation of the TCR and CD5, which can be used to monitor thymocyte activation and maturation (23, 24). As shown in **Figure 7A**, the levels of TCRβ and CD5 expression were similar among DP, CD4SP, and CD8 SP cell populations when comparing Paxbp1 cKO mice with control mice. According to the expression patterns of TCRβ and CD69 (a T cell maturation marker (25),), the four different stages can be divided to visualize DP development. There was no difference in the proportion of four stages between the cKO and control groups (**Figures 7B, C**). In addition, gating on the CD4 SP or CD8 SP population, the proportions of immature SP cells (TCRβ^hi^CD69^hi^) and mature SP cells (TCRβ^hi^CD69^low/-^) were similar between control and Paxbp1 cKO mice (**Figures 7B, C**). Thus, Paxbp1 deficiency did not alter TCRβ, CD5, or CD69 expression in DP and SP cell populations, suggesting that T cells mature normally in Paxbp1 cKO mice.

### 3.7 Reduced peripheral T cell numbers in Paxbp1 cKO mice

**Figure 8:**
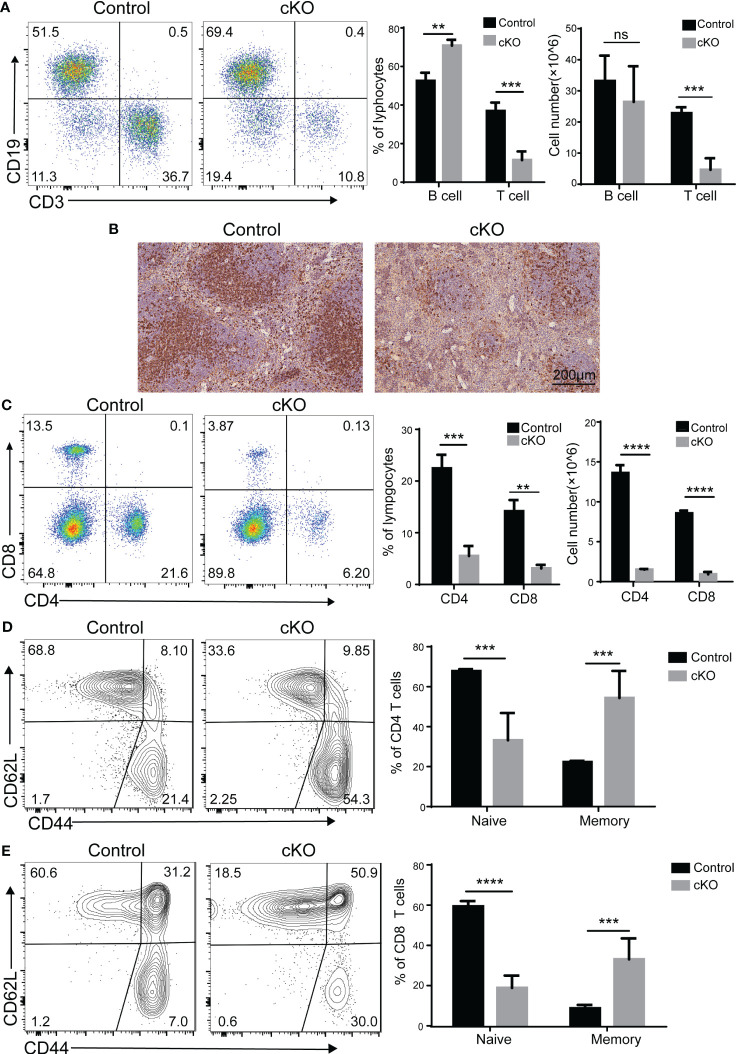
Phenotype of peripheral T cells in Paxbp1 cKO mice. **(A)** Cell surface staining of CD3 and CD19 on control and Paxbp1 cKO splenocytes; bar charts indicate the frequency and cell number of T cells and B cells in the spleen (n > 3 for each genotype). **(B)** CD4 levels were analyzed in the control and Paxbp1 cKO spleen samples using immunohistochemistry (Scale bar = 200 µm). **(C)** Cell surface staining of CD4 and CD8 on control and Paxbp1 cKO splenocytes; bar charts indicate frequency and cell number of subsets in the spleen (n > 4 for each genotype). **(D)** Expression of CD62L and CD44 on CD4^+^ splenocytes from the control and Paxbp1 cKO mice; bar chart shows the frequency of CD4^+^ cells in the CD62L^hi^CD44^lo^ (naïve T cell) and CD62L^lo^CD44^hi^ (memory T cell) subpopulations. **(E)** Expression of CD62L and CD44 on CD8^+^ splenocytes from the control and Paxbp1 cKO mice; bar chart shows the frequency of CD8^+^ cells in the CD62L^hi^CD44^lo^ (naïve T cell) and CD62L^lo^CD44^hi^ (memory T cell) subpopulations. The p value was used to determine whether an effect was significant (** p < 0.01; *** p <0.001; **** p < 0.0001; ns means significant).

Since CD4^+^CD8^+^ double-positive (DP) thymocyte survival is crucial in shaping the peripheral T cell repertoire (26), we investigated whether the peripheral T-cell population was affected by the decreased thymocytes in Paxbp1 cKO mice. As expected, the B cells in the spleen were not affected, whereas fewer peripheral T cells were seen in the spleen of Paxbp1-deficient mice (**Figure 8A**). Meanwhile, immunohistochemical staining of spleen sections from Paxbp1 cKO mice showed significantly reduced CD4 expression compared to the control mice (**Figure 8B**). Consistent with this result, Paxbp1 deficiency led to fewer CD4^+^ and CD8^+^ T cells in the spleen (**Figure 8C**). Interestingly, Paxbp1 cKO animals displayed higher frequencies of effector/memory (CD44^hi^ CD62L^lo^) cells in their splenic CD4^+^ or CD8^+^ T cell populations than control mice (**Figures 8D, E**), which is common in lymphopenic mice and indicates homeostatic expansion. Taken together, these findings reveal that poor thymocyte formation in Paxbp1 cKO mice leads to peripheral lymphopenia.

## 4 Discussion

T cell development in the thymus is a highly organized biological process that combines lineage commitment, differentiation, proliferation, death, and selection (27, 28). As a conserved nuclear protein, Paxbp1 is highly expressed in T cells and has been reported to play important roles in the development of multiple tissues. However, its role in T cell development is poorly studied. Here, we provided novel evidence for an important and non-redundant role for the Paxbp1 protein during thymocyte development. The Lck-cre-mediated knockout of Paxbp1 in T cells caused thymic atrophy and decreased thymocyte number, particularly from the DP stage onwards (**Figures 1**, **2**). However, Paxbp1 deficiency exerted limited effects on the DN and ISP cell populations, suggesting that Paxbp1 mainly impacts thymus development at the DP cell stage (**Figures 3**, **4**).

Cell proliferation and apoptosis are necessary for thymocyte development and homeostasis (29). We therefore examined the proliferative and apoptotic properties of DP thymocytes in our Paxbp1 cKO mouse model. Our results indicate increased *in vivo* and *in vitro* apoptosis in DP thymocytes from Paxbp1 cKO mice compared to control mice (**Figure 4**). In addition, Paxbp1-deficient thymocytes are highly sensitive to apoptosis caused by anti-CD3 antibody treatment, as supported by increased annexin V labeling and enhanced caspase-3 activity in Paxbp1-deficiency DP thymocytes after anti-CD3 antibody treatment (**Figure 4**). These data point to abnormal regulation of programmed cell death in DP thymocytes in the absence of Paxbp1.

Interestingly, higher BrdU incorporation was also found in Paxbp1 cKO DP thymocytes (**Figure 6**), indicating that the decrease in thymocyte counts in Paxbp1 cKO thymus might be due to lower DP cell survival rather than a deficiency in proliferation. This observation agrees with previous data showing that enhanced thymocyte apoptosis is accompanied by an increase in the number of circulating thymocytes (30).

Here, RNA-Seq was used to identify differentially expressed genes between DP thymocytes from control and Paxbp1 cKO mice, which may function as components of the cellular death pathway. According to the RNA-seq result (**Figure 5**), p53 signaling and the apoptosis pathway were activated, and the expression of several cell death-related genes such as *Bim*, *Pmaip1*, *Pidd1*, and *Casp3* was significantly increased. The pro-apoptotic protein Bim belongs to the B cell lymphoma 2 (BCL-2) family and has integral roles in the development and function of the immune system (31). Thymocytes lacking Bim are resistant to apoptosis induced by CD3 stimulation and interleukin-2 (IL-2) withdrawal (which induces apoptosis) (32, 33). Like Bim, Pmaip1 is a BH3-only protein of the Bcl-2 family that is important for the initiation of apoptotic cell death in response to DNA damage (34–36). PIDD (P53-induced protein with a death domain), encoded by *Pidd1*, also participates in DNA damage-induced apoptosis. Moreover, it has been described as an apoptosis-inducing protein acting *via* activating caspase-2 (33, 37). Thus, increased Bim, Pmaip1, and Pidd1 levels caused by Paxbp1 knockout may explain the increased apoptosis in the thymocytes of Paxbp1 cKO mice. However, it remains to be seen whether the increased expression of these proteins in Paxbp1 deficient thymocytes contributes directly to their higher sensitivity to apoptosis.

The reduction in thymic T-cell production strongly influences the peripheral T-cell population. As expected, the proportion of T cells in peripheral immune organs in Paxbp1 cKO mice decreased significantly relative to controls (**Figure 8**). In addition, we found a lower proportion of naïve T cell populations and a higher proportion of memory T cell populations in Paxbp1 cKO mice (**Figure 8**), indicating homeostatic expansion. As Lck-cre-mediated, T-cell-specific Paxbp1 deletion leads to marked thymic atrophy and the reduction in thymic T-cell production has a great effect on the peripheral T-cell population, we do not think this model is optimal for studying the effect of Paxbp1 on peripheral T-cells.

Here, we have characterized the effect of Paxbp1 deficiency on thymic development, which mainly results in reduced thymic cellularity as a result of decreased DP cell survival. Mechanistically, the developmental defect in DP thymocytes may be due to activation of the apoptotic pathway. Our data provide novel evidence for the indispensable role of Paxbp1 in T cell development. This discovery could aid in developing more effective plans for immune reconstitution in people with immunodeficiency.

## Data availability statement

The original contributions presented in the study are included in the article/**Supplementary Material**. Further inquiries can be directed to the corresponding authors. All sequencing data have been submitted to the NCBI Gene Expression Omnibus (GEO; http://www.ncbi.nlm.nih.gov/geo/) under accession number GSE233211.

## Ethics statement

The animal study was reviewed and approved by Committee for the Ethics of Animal Experiments, Shenzhen Peking University‐The Hong Kong University of Science and Technology Medical Center (SPHMC). Written informed consent was obtained from the owners for the participation of their animals in this study.

## Author contributions

WL contributed to the experimental design, performance, data collection/analysis, and manuscript preparation. YY, SL, DZ contributed to experimental design and data analysis. XR and MT helped to data analysis. CH contributed to the data analysis and manuscript preparation. WZ, XC, and BY contributed to conception and design of the study. All authors contributed to the article and approved the submitted version.
