# Supplementary material for: Paxbp1 is indispensable for the survival of CD4 and CD8 double-positive thymocytes
Source: Front Immunol. 2023 Jun 19;14:1183367. doi: 10.3389/fimmu.2023.1183367 (PMC10315898; doi:10.3389/fimmu.2023.1183367)
Supplement: Supplementary file 1 [file DataSheet_1.docx]

Supplementary Material

Paxbp1 is indispensable for the survival of CD4 and CD8 double-positive thymocytes

Wenting Li^1,2^, Yang Yang^3^, Shenglin Liu^4^, Dongsheng Zhang^3^, Xuanyao Ren^3^, Mindan Tang^3^, Wei Zhang^3,5^, Xiaofan Chen^3*†^, Cong Huang^1,2*†^, Bo Yu^1,2*†^

^1^Department of Dermatology, Peking University Shenzhen Hospital, Shenzhen, Guangdong, China.

^2^Shenzhen Key Laboratory for Translational Medicine of Dermatology, Shenzhen Peking University - The Hong Kong University of Science and Technology Medical Center, Shenzhen, Guangdong, China.

^3^Shenzhen Key Laboratory for Translational Medicine of Dermatology, Biomedical Research Institute, Shenzhen Peking University - The Hong Kong University of Science and Technology Medical Center, Shenzhen, Guangdong, China.

^4^Key Laboratory of Research and Utilization of Ethnomedicinal Plant Resources of Hunan Province, College of Biological and Food Engineering, Huaihua University, Huaihua, Hunan, China.

^5^Shenzhen Bay Laboratory, Shenzhen, Guangdong, China.

*** Correspondence:**

These authors contributed equally to this work and share last authorship.

Dr. Xiaofan Chen, Shenzhen Key Laboratory for Translational Medicine of Dermatology, Biomedical Research Institute, Shenzhen Peking University - The Hong Kong University of Science and Technology Medical Center, Shenzhen, Guangdong Province, China. Email: [littlecanva@163.com](mailto:littlecanva@163.com)

Dr. Cong Huang, Department of Dermatology, Peking University Shenzhen Hospital, Shenzhen, Guangdong Province, China. Email: [conghuangphd1988@163.com](mailto:conghuangphd1988@163.com)

Dr. Bo Yu, Department of Dermatology, Peking University Shenzhen Hospital, Shenzhen, Guangdong Province, China. Email: drboyu_derm@126.com

# Supplementary Figures


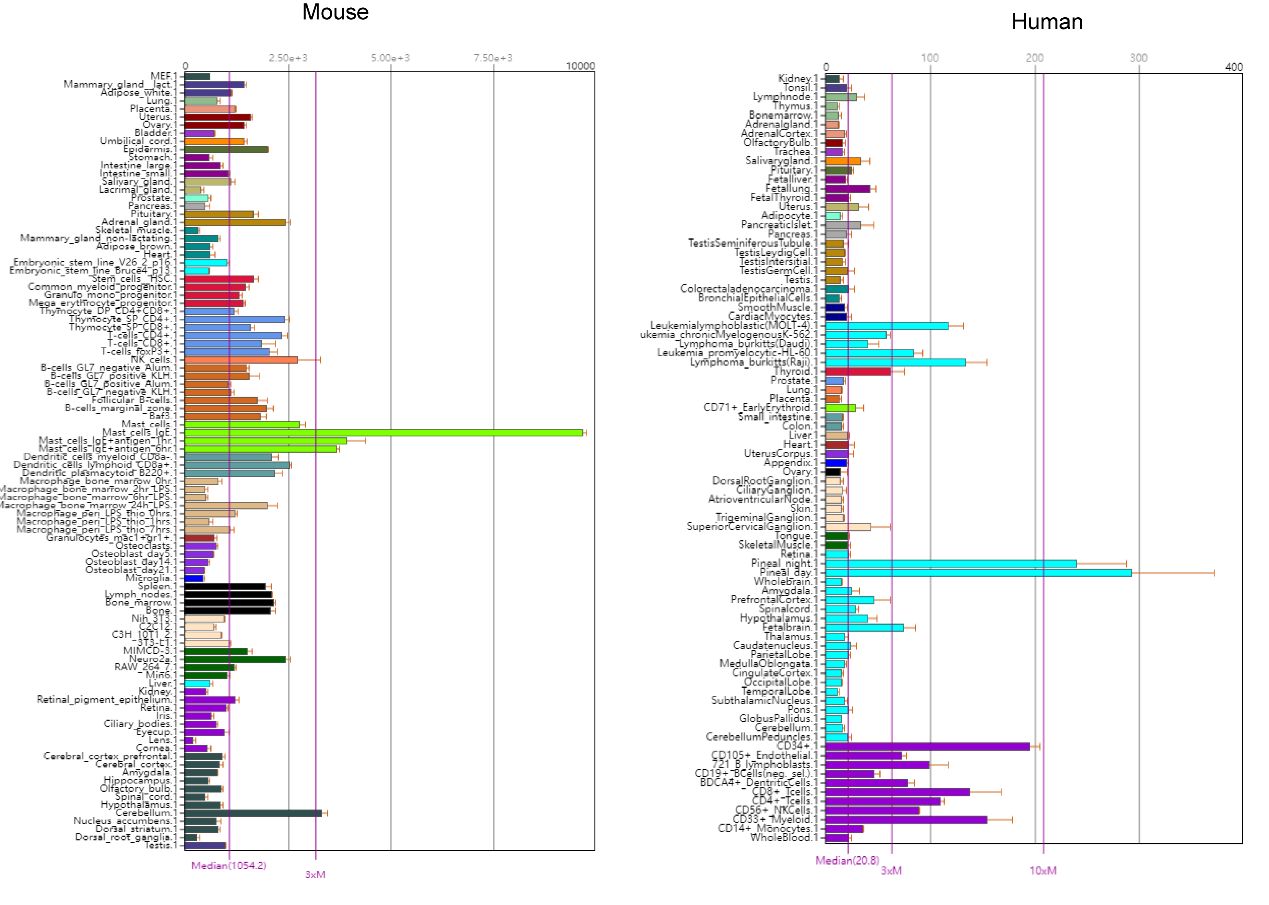


**Supplementary Fig1**. Expression of Paxbp1 in different tissues and cells of mice and humans, data from BioGPS database.
